# Effects of the Traditional Chinese Medical Prescription Linba Fang as a Treatment for Lymphedema

**DOI:** 10.1155/2020/8889460

**Published:** 2020-11-12

**Authors:** Y. Luo, L. Zhao, N. F. Liu

**Affiliations:** ^1^Department of Plastic and Reconstructive Surgery, Shanghai Ninth People's Hospital, Shanghai JiaoTong University School of Medicine, Shanghai 200011, China; ^2^School of Pharmacy, Shanghai JiaoTong University, Shanghai 200240, China

## Abstract

Lymphedema can lead to a series of complicated and irreversible chronic pathological changes, including lymphatic fluid retention, infiltration of inflammatory cells, lipid deposition, and fibrosis of the surrounding tissues. Typically, compression physiotherapy is recommended for early lymphedema. However, the chronic fluid compartments will lead to fat deposition, skin fibrosis, and hyperkeratosis. Few treatment methods are available for patients with lymphedema. Previous studies have attempted to apply diuretics, diosmin, and sodium *β*-aescinate to treatment for venous edema, but the curative effect was unsatisfactory. There is currently no established effective treatment for lymphedema. In this paper, we investigated the effects of the traditional Chinese medical prescription Linba Fang as a treatment for lymphedema using a mouse model. A lymphedema model was established in C57BL/6 mice through lymphatic ablation at the base of tails. Negative controls were administered with 0.5% sodium carboxymethyl cellulose solution by gavage twice daily, positive controls with aescuvenforte, and test mice with Linba Fang. Aescuvenforte and Linba Fang were dissolved in 0.5% sodium carboxymethyl cellulose solution to produce a homogeneous mixture. After treatment for 2–4 weeks, tail diameter and weight, inflammatory cytokine levels (IL-1, IL-6, and TNF-*α*), lipid deposition, and fibrosis were evaluated. The results showed that none of the mice died during the treatment with Linba Fang. The levels of tail swelling, inflammation, lipid deposition, and fibrosis in mice treated with Linba Fang were significantly decreased compared with negative and positive controls. Among mice treated with the same dose of Linba Fang, the levels of tail swelling, inflammation, lipid deposition, and fibrosis in mice treated for 4 weeks were significantly lower than those treated for 2 weeks. Among mice treated for the same duration of time, the levels of tail swelling, inflammation, lipid deposition, and fibrosis showed a decreasing tendency following increasing doses. Notably, the inflammation in tail tissues decreased to the similar level of normal group after treatment for 4 weeks using the high dose of Linba Fang. In conclusion, the traditional Chinese medical prescription Linba Fang could inhibit the pathological changes caused by lymphedema, including swelling, inflammation, lipid deposition, and fibrosis.

## 1. Introduction

The lymphatic system is the secondary circulatory system of the body and plays a critical role in transport of lipids, regulation of body fluid homeostasis, and immune cell trafficking [1]. The lymphatic system collects fluids and proteins from the interstitial space and returns them to the blood circulation [2, 3]. However, lymph stasis induced by both congenital and postnatal abnormalities disrupts the normal conduit for the return of interstitial fluids and proteins to the blood circulation and causes an accumulation of proteins and cellular metabolites in the extracellular space, resulting in an ensuing increase in tissue colloid osmotic pressure, water accumulation, and elevation of the interstitial hydraulic pressure [4]. Lymphedema is a disorder caused by lymphatic obstruction associated with lymphatic injury or disease. Lymphedema can lead to a series of complicated and irreversible chronic pathological changes, including lymphatic fluid retention, infiltration of inflammatory cells, lipid deposition, and fibrosis of the surrounding tissues [5]. Typically, compression physiotherapy is recommended for early lymphedema. However, the chronic fluid compartments will lead to fat deposition, skin fibrosis, and hyperkeratosis [1]. In addition, lymphangitis and inflammation of surrounding tissues (cellulitis) become gradually more frequent following lymphedema. Infections, in turn, aggravate lymphedema, resulting in a vicious cycle of disease [6].

Few treatment methods are available for patients with lymphedema. Previous studies have attempted to apply diuretics, diosmin, and *β*-aescinate sodium (horse chestnut seed extract) to treatment for venous edema, but the curative effect was unsatisfactory [6, 7]. There is currently no established effective treatment for lymphedema. Therefore, it is urgent to develop a more effective therapy to reduce tissue edema and relieve complications associated with lymphedema, including infection and tissue inflammation. A safe and effective treatment for lymphedema and its complications was developed by the Center for Lymphedema Treatment of the Shanghai Ninth People's Hospital in 2009, which used the traditional Chinese medical compound prescription Linba Fang. The aim of this study was to investigate the effects of the new method in the treatment of lymphedema through a mouse model.

## 2. Materials and Methods

### 2.1. Animals

C57BL/6 mice were purchased from the Shanghai Silaike Experiment Animals Co., Ltd. A total of 110 male C57BL/6 mice aging 10–12 weeks, with a weight of (20 ± 2) g, were included in this study. All mice were kept at 20∼25°C and 50%∼60% of relative humidity in a specific pathogen-free (SPF) animal room with free access to water and food. All experimental procedures were approved by the Institutional Animal Care and Use Committee of Shanghai Jiao Tong University (SJTU-IACUC-AP-20140827001).

### 2.2. Establishment of a Mouse Model for Lymphedema

A lymphedema model was established in mice through lymphatic ablation at the base of tails. The mice were administered with 0.01 mL of 0.1% methylene blue in the tail. A circumferential incision was made in the deep fascia (removing 2 mm of skin) close to the base of the tail with the aid of a dissecting microscope, and the skin along the incision was removed, exposing a 2-3 mm-wide wound. Blue-stained lymphatic vessels were excised and ligated, after which electrocoagulation and cauterization were used to electrocauterize the visible lymphatic capillaries and the skin at the edge of the wound. The wounds were left to natural healing. The animal model was established after 3 months of feeding. Indocyanine green (ICG) was injected into the tail of each mouse as a contrast agent. The lymphedema model was successfully established when the development of ICG contrast was confirmed, i.e., the unabsorbed contrast agent was prevented from flowing back into the lymphatic circulation due to the damaged lymphatic vessels in the tail.

### 2.3. Grouping

The mice were divided into 11 groups, and grouping and corresponding treatment were demonstrated in Table 1. The lymphedema model was established in all mice except for the normal group. The test drugs (aescuvenforte or Linba Fang) were administered by gavage twice daily in all mice except for the normal group. The drugs were dissolved in 0.5% sodium carboxymethyl cellulose solution to produce a homogeneous mixture. Negative control groups (model groups) were administered with 0.5% sodium carboxymethyl cellulose solution because Linba Fang was dissolved with sodium carboxymethyl cellulose.

### 2.4. Composition of Linba Fang

In this study, a Chinese herbal medicine compound prescribe, called Linba Fang, was used in the treatment of lymphedema in a mouse tail model. Linba Fang consisted of 2 traditional Chinese herbal medicines including Sophora flavescens Ait and *Salvia miltiorrhiza*. The extraction content of Tanshinone IIA from *Salvia miltiorrhiza* was 7 mg/g, and the extraction content of matrine from Sophora flavescens Ait was 3 mg/g.

### 2.5. Measurement of Tail Diameter and Weight

The diameter of the lymphatic obstruction area of each mouse was measured at 3 points: the base of the tail, 10 mm from the base of the tail, and 20 mm from the base of the tail (designated as P, P10, and P20, respectively). A vernier caliper was used for all measurements. After the mice were sacrificed, the tails were removed proximal to the wound site (designated as P) for measurement of tail weight. Each tail was weighed 3 times to obtain an average value.

### 2.6. Quantification of Inflammatory Cytokines in Serum and Tail Tissue Homogenate

Blood samples collected from the orbital venous plexus of the mice were centrifuged in anticoagulant tubes to obtain serum, which was stored at −80°C. Tails of 1 cm from the incision site were excised from the sacrificed mice and frozen in liquid nitrogen prior to grinding. Normal saline was added at a weight ratio of 1 : 10, followed by homogenization in a homogenizer. The resulting homogenate samples were centrifuged to obtain supernatant samples, which were stored at −80°C. Enzyme-linked immunosorbent assay (ELISA) was used to measure the levels of IL-1*β*, IL-6, and TNF-*α* in serum and tail tissue homogenate with a BioTek ELx800 ELISA reader (BioTek Co., LTD, USA).

### 2.7. Masson's Trichrome Staining and Oil Red O Staining of Mouse Tail Tissue

Frozen sectioning was performed on the tissue obtained from the mouse tail proximal to the wound site (designated as P). For Masson's trichrome staining, nuclear staining was performed on the tissue sections with hematoxylin for 5 minutes, followed by rinsing with distilled water and differentiation using HCl-alcohol. The tissue sections were stained with Ponceau-acid Fuchsin solution for 5 min and immersed in 2% glacial acetic acid solution for about 30 s, followed by differentiation in 1% phosphomolybdic acid solution and rinsing with 0.2% glacial acetic acid solution. The tissue sections were immersed in Brilliant Green solution for 5 min and immersed in 2% glacial acetic acid solution for 30 s, followed by immersion in 95% alcohol, 100% alcohol, and xylene for 1 min, respectively. Finally, the tissue sections were mounted in neutral resin.

For Oil Red O staining, the dried, frozen tissue sections were rehydrated in 50% ethanol, followed by immersion in Oil Red O working solution for 8 min. The tissue sections were removed from the staining solution and rinsed with tap water, followed by nuclear staining with hematoxylin. Finally, the samples were rinsed with tap water and mounted in gelatin.

### 2.8. Western Blots

Tails of 1 cm from the incision site were collected from sacrificed mice. Total protein was extracted from subcutaneous tail tissue after separation of the tissue from the bone. For protein extraction, 5 mm full-thickness tissue sections were harvested, frozen in liquid nitrogen, and homogenized. Total cellular protein was extracted using the Tissue Protein Extraction Reagent (T-PER, ThermoFisher Scientific, Rockford, IL) and quantified using the Bradford technique (Bio-Rad Protein Assay, Hercules, CA). Proteins (4–6 *μ*g) were separated on 10% SDS-polyacrylamide gel by electrophoresis (1 × Tris-glycine/0.1% SDS buffer) and transferred to a polyvinylidene fluoride membrane (Bioexpress, Kaysville, UT). Membranes were blocked using 5% nonfat milk in 1 × Tris-buffered Saline Tween 20 (TBST) and incubated with primary antibodies against CEBP-*α*, PPAR-*γ*, Type I collagen, or adiponectin (Abcam, Cambridge, MA) followed by appropriate HRP-conjugated secondary antibody (Santa Cruz Biotechnology, Santa Cruz, CA). Staining was detected by using the ECL Plus Western Blotting Detection System (GE Healthcare, Little Chalfont, UK). Loading levels were equalized by *β*-actin (Abcam) staining. Experiments were repeated in triplicate, and ImageJ software used to determine relative signal densities after normalization to actin as previously described.

### 2.9. Statistical Analysis

Statistical analysis was conducted using the SPSS version 21 (IBM, Armonk, NY, USA). One-way ANOVA was employed to perform comparison among groups with the Student–Newman–Keuls method. Significance was set at two-sided *P* < 0.05.

## 3. Results

### 3.1. Tail Diameters and Weights

**Figure 1:**
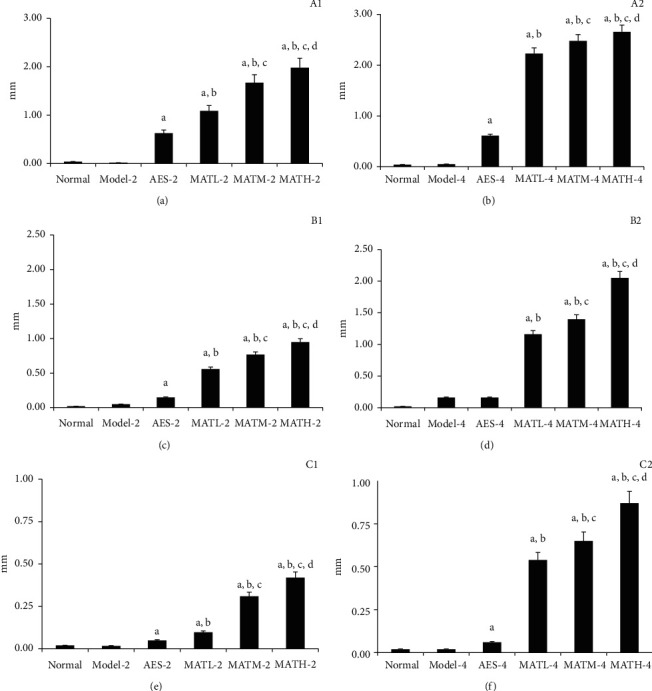
Decreased levels of tail diameters at 3 points after treatment. (a) 2 weeks after treatment at point *P*, (b) 4 weeks at point *P*, (c) 2 weeks at point P10, (d) 4 weeks at point P10, (e) 2 weeks at point P20, and (f) 4 weeks at point P20. a: *P* < 0.05 vs. model groups, b: *P* < 0.05 vs. AES groups, c: *P* < 0.05 vs. MATL groups, and d: *P* < 0.05 vs. MATM groups.

None of the mice died during the treatment with Linba Fang. The decreased levels of tail diameters following treatment are demonstrated in Figure 1. The decreased levels of mice treated with Linba Fang were significantly greater than those of negative and positive controls. Among mice treated with the same dose of Linba Fang, the decreased levels of mice treated for 4 weeks were significantly greater than those of mice treated for 2 weeks (*P* < 0.05). Among mice treated for the same duration of time, the decreased levels demonstrated an increasing tendency following increasing doses. There was no significant difference in the decreased level between negative control and normal groups.

**Figure 2:**
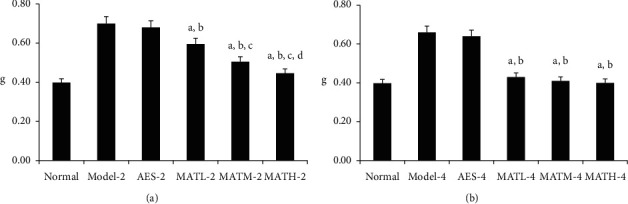
Tail weights AFTER treatment. (a) 2 weeks after treatment and (b) 4 weeks after treatment. a: *P* < 0.05 vs. model groups, b: *P* < 0.05 vs. AES groups, c: *P* < 0.05 vs. MATL groups, and d: *P* < 0.05 vs. MATM groups.

As shown in Figure 2, the tail weights of mice treated with Linba Fang were significantly less than those of negative and positive controls. Among mice treated with the same dose of Linba Fang, the tail weights of mice treated for 4 weeks were significantly less than those of mice treated for 2 weeks (*P* < 0.05). Among mice treated for 2 weeks, the tail weights demonstrated a decreasing tendency following increasing doses, but there was no significant difference among mice treated for 4 weeks.

### 3.2. Inflammatory Cytokines in Serum and Tail Tissue Homogenate

**Figure 3:**
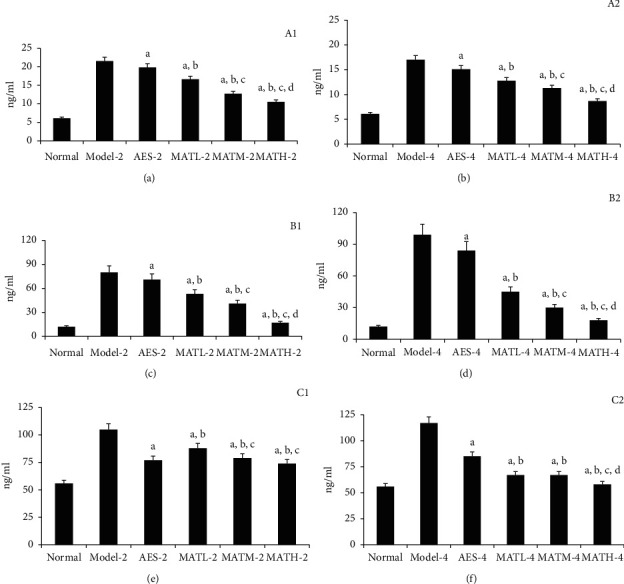
Inflammatory cytokine levels in serum after treatment. (a) IL-1 for 2 weeks after treatment, (b) IL-1 for 4 weeks, (c) IL-6 for 2 weeks, (d) IL-6 for 4 weeks, (e) TNF-*α* for 2 weeks, and (f) TNF-*α* for 4 weeks. a: *P* < 0.05 vs. model groups, b: *P* < 0.05 vs. AES groups, c: *P* < 0.05 vs. MATL groups, and d: *P* < 0.05 vs. MATM groups.

As shown in Figure 3, IL-1, IL-6, and TNF-*α* levels in serum of mice treated with Linba Fang were significantly lower than those of negative and positive controls. Among mice treated with the same dose of Linba Fang, IL-1, IL-6, and TNF-*α* levels of mice treated for 4 weeks were significantly lower than those of mice treated for 2 weeks (*P* < 0.05). Among mice treated for the same duration of time, IL-1 and IL-6 levels demonstrated a decreasing tendency following increasing doses (*P* < 0.05). IL-1 and IL-6 levels of mice treated with Linba Fang were significantly higher than that of the normal group. TNF-*α* levels of mice treated with Linba Fang for 2 weeks were significantly higher than that of the normal group, but TNF-*α* decreased to the similar level of normal group after treatment for 4 weeks using the high dose.

**Figure 4:**
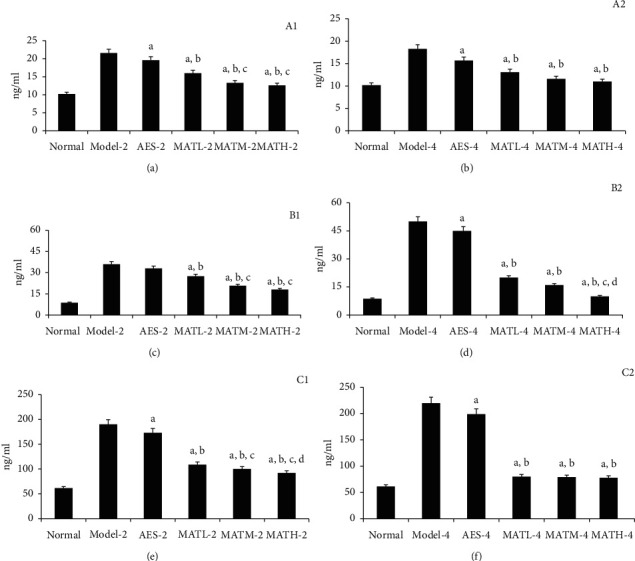
Inflammatory cytokine levels in tail tissue homogenate after treatment. (a) IL-1 for 2 weeks after treatment, (b) IL-1 for 4 weeks, (c) IL-6 for 2 weeks, (d) IL-6 for 4 weeks, (e) TNF-*α* for 2 weeks, and (f) TNF-*α* for 4 weeks. a: *P* < 0.05 vs. model groups, b: *P* < 0.05 vs. AES groups, c: *P* < 0.05 vs. MATL groups, and d: *P* < 0.05 vs. MATM groups.

As shown in Figure 4, IL-1, IL-6, and TNF-*α* levels in tail tissue homogenate of mice treated with Linba Fang were significantly lower than those of negative and positive controls. Among mice treated with the same dose of Linba Fang, IL-6 levels of mice treated for 4 weeks were significantly lower than those of mice treated for 2 weeks (*P* < 0.05). Among mice treated with Linba Fang for 2 weeks, IL-1, IL-6, and TNF-*α* levels demonstrated a decreasing tendency following increasing doses. Among mice treated with Linba Fang for 4 weeks, IL-6 levels demonstrated a decreasing tendency following increasing doses. IL-1, IL-6, and TNF-*α* levels of mice treated with Linba Fang for 2 weeks were significantly higher than those of the normal group, but IL-1, IL-6, and TNF-*α* decreased to the similar levels of the normal group after treatment for 4 weeks using the high dose.

### 3.3. Lipid Deposition in Tail Tissues

#### 3.3.1. Staining of Tail Tissues with Oil Red O

**Figure 5:**
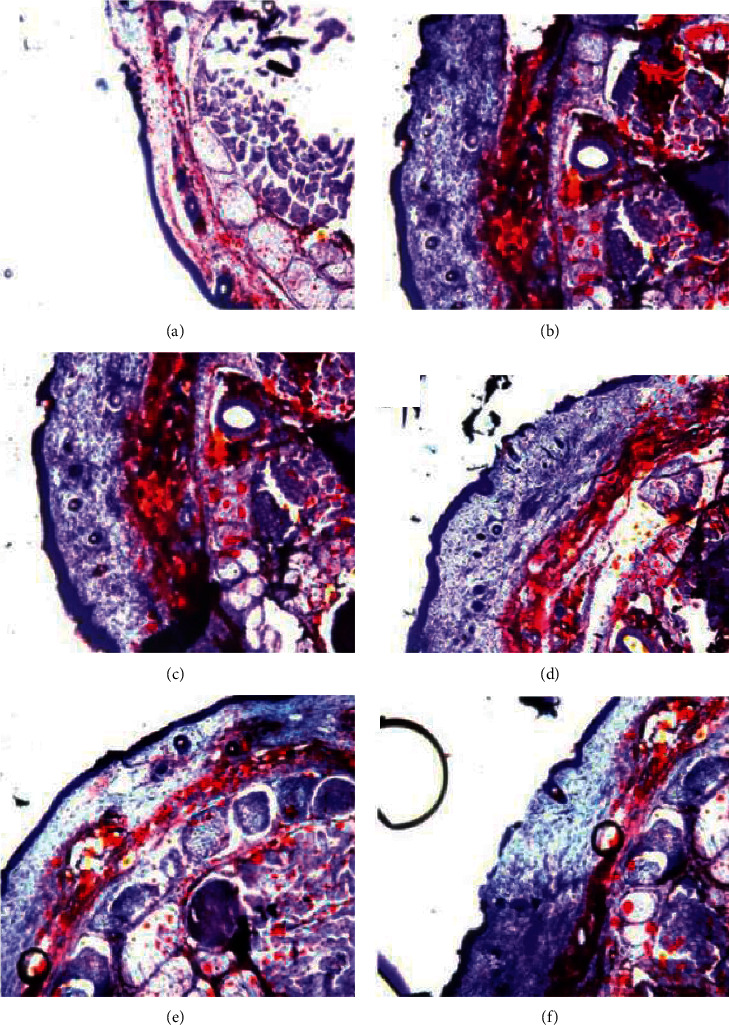
Subcutaneous fat thickness of tails after treatment. (a) Normal group, (b) model 4 group, (c) AES-4 group, (d) MATL-4 group, (e) MATM-4 group, and (f) MATH-4 group.

The subcutaneous fat thickness of tail tissues in mice treated with Linba Fang was decreased compared with that of negative and positive controls. Among mice treated with Linba Fang, the subcutaneous fat thickness demonstrated a decreasing tendency following increasing doses. Negative and positive controls had thicker subcutaneous fat compared with normal group (Figure 5).

#### 3.3.2. PPAR-*γ* and CEPB-*α* Levels in Tail Tissues

**Figure 6:**
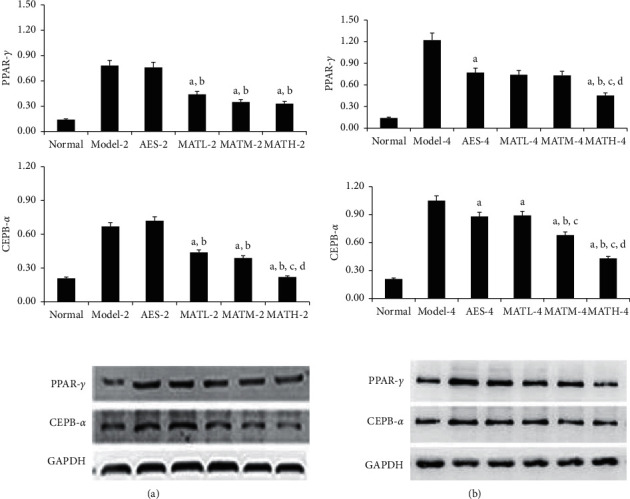
PPAR-*γ* and CEPB-*α* levels in tail tissues after treatment. (a) 2 weeks after treatment and (b) 4 weeks after treatment. a: *P* < 0.05 vs. model groups, b: *P* < 0.05 vs. AES groups, c: *P* < 0.05 vs. MATL groups, and d: *P* < 0.05 vs. MATM groups.

PPAR-*γ* and CEPB-*α* are regulatory proteins upstream and downstream, respectively, of lipid deposition. The expression levels of PPAR-*γ* and CEPB-*α* are indicative of the degree of lipid deposition. As shown in Figure 6, PPAR-*γ* and CEPB-*α* levels in tail tissue from negative and positive controls were significantly higher than those of the normal group. PPAR-*γ* and CEPB-*α* levels of tail tissue in mice treated with Linba Fang were significantly lower than those of negative and positive controls. Among mice treated for the same duration of time, PPAR-*γ* and CEPB-*α* levels showed a decreasing tendency following increasing doses.

### 3.4. Fibrosis in Tail Tissues

#### 3.4.1. Masson's Trichrome Staining of Tail Tissues

**Figure 7:**
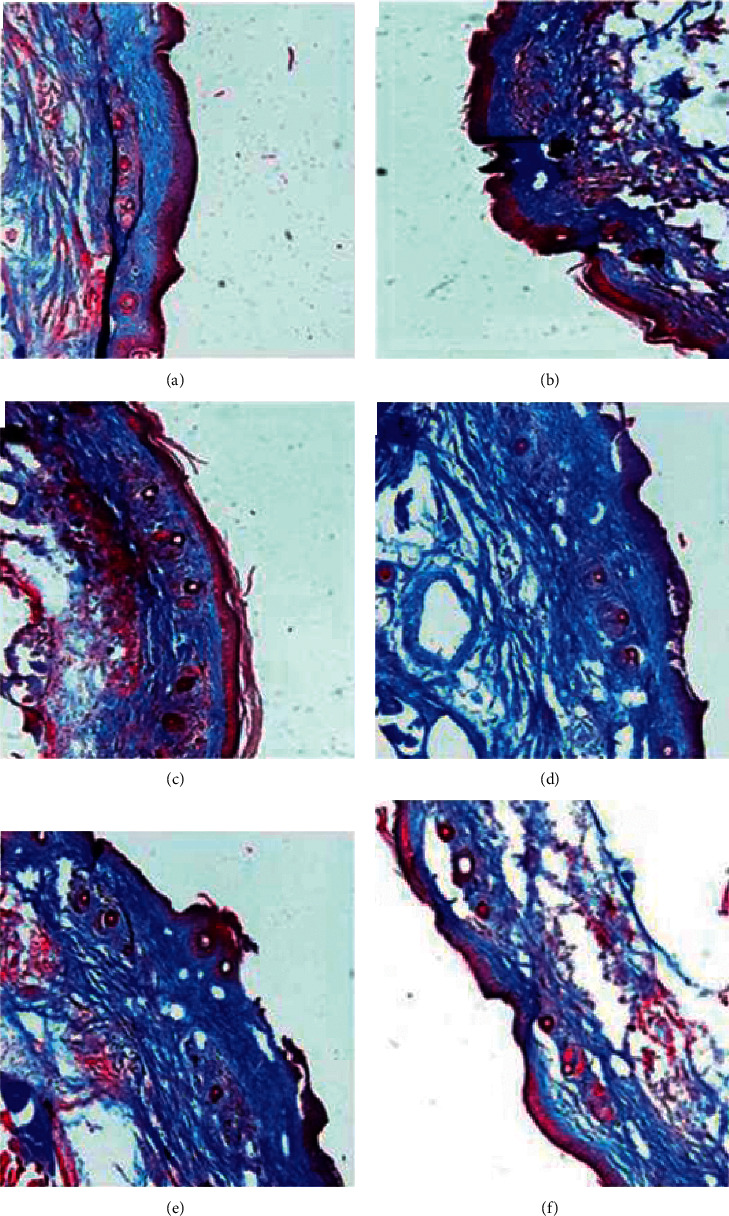
Subcutaneous fibrous layer of tail tissues after treatment. (a) Normal group, (b) model 4 group, (c) AES-4 group, (d) MATL-4 group, (e) MATM-4 group, and (f) MATH-4 group.

The subcutaneous fibrous layer of tail tissues in negative and positive controls was thicker than that in the normal group. The subcutaneous fibrous layer in mice treated with Linba Fang was thinner than that in negative and positive controls. Among mice treated with Linba Fang for 4 weeks, the thickness of the subcutaneous fibrous layer showed a decreasing tendency following increasing doses (Figure 7).

#### 3.4.2. Type I Collagen Levels in Tail Tissues

**Figure 8:**
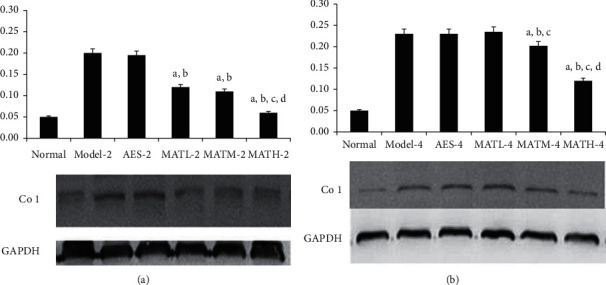
Type I collagen levels in tail tissues after treatment. (a) 2 weeks after treatment and (b) 4 weeks after treatment. a: *P* < 0.05 vs. model groups, b: *P* < 0.05 vs. AES groups, c: *P* < 0.05 vs. MATL groups, and d: *P* < 0.05 vs. MATM groups.

The levels of Type I collagen reflect the degree of dermal fibrosis. As shown in Figure 8, Type I collagen levels of negative and positive controls were higher than those of the normal group, and Type I collagen levels of mice treated with Linba Fang were lower than those of negative and positive controls. Among mice treated for the same duration of time, Type I collagen levels showed a decreasing tendency following increasing doses.

## 4. Discussion

Lymphedema is the consequence of an imbalance between the rate of lymph production and its removal through the lymphatic vascular channels, which can lead to a series of complex pathological changes, including edema, accumulation of macromolecules, chronic inflammation, fibrosis, and lipid deposition [8, 9]. Acquired lymphedema is the most commonly encountered form of lymphatic dysfunction. The clinical characteristics of lymphedema include acute and chronic inflammation, lipid deposition, and fibrosis [8, 9]. Studies using models of lymphedema in the mouse tail have also found that, in addition to edema, subcutaneous lipid deposition is a symptom of lymphedema. Additionally, collagen also appears to accumulate at the region of lipid deposition, leading to fibrosis. Chronic lymphedema can lead to dermal and subcutaneous fibrosis, as well as fibrosis of remaining lymphatic capillaries [10]. Lipid deposition is closely associated with inflammation [11, 12], which in turn further aggravates the symptoms of lipid deposition and fibrosis [13]. If lymphedema is not properly treated during its early stage, the pathological changes described above develop, eventually leading to severe deformity and limb dysfunction.

Despite continuing developments of treatment approaches, the treatment options of acquired lymphedema remain limited. The application of pharmacological therapies has been notably absent from the management strategies for lymphatic vascular insufficiency states. Previous studies have reported the application of aescuvenforte (sodium *β*-aescinate), diosmin, and other drugs in the treatment of lymphedema. Sodium *β*-aescinate and diosmin are mainly used to treat chronic venous insufficiency (ICV), as well as soft tissue swelling and venous edema caused by various factors, but they are ineffective in patients with pure lymphedema [14–18]. No drugs are effective against all of the major pathological changes associated with lymphedema. The effects of drugs against particular pathological symptoms of lymphedema are generally limited. Therefore, treatment targeting at the multiple basic pathological changes associated with lymphedema is needed to significantly relieve the symptoms of lymphedema.

In this study, a Chinese herbal medicine compound prescribe, called Linba Fang, was used in the treatment of lymphedema in a mouse tail model. Linba Fang consisted of 2 traditional Chinese herbal medicines including Sophora flavescens Ait and *Salvia miltiorrhiza*. We have found that Linba Fang treatment exhibits significant effects on the complex pathological changes associated with lymphedema compared with positive controls treated with aescuvenforte. Our study showed that the levels of inflammatory cytokines (IL-1, IL-6, and TNF-*α*) in serum and tail tissues of mice treated with were significantly lower than those of negative and positive controls. Among mice treated with the same doses of Linba Fang, the levels of inflammatory cytokines of mice treated for 4 weeks were significantly lower than those of mice treated for 2 weeks. Among mice treated for the same durations of time, the levels of inflammatory cytokines showed a decreasing tendency following increasing doses. These results suggested that Linba Fang had significant effects of antisepsis and anti-inflammation. This may be explained by Sophora flavescens Ait which is the main component of Linba Fang and contains large amounts of matrine with strong antibacterial effects. In addition, *Salvia miltiorrhiza*, another component of Linba Fang, has antibacterial, anti-inflammatory, and hormone-like effects [19].

Platelet-derived growth factor-BB (PDGF-BB) plays an important role in fibroblast proliferation, while transforming growth factor-beta 1 (TGF-*β*1) plays an important role in collagen synthesis [20]. Both of them are critical to the regulation of dermal fibrosis. Matrine has strong inhibitory effects on PPDF-BB-induced fibroblast proliferation and TGF-*β*1-induced collagen synthesis and, therefore, effectively alleviates dermal fibrosis. In this study, we found that the subcutaneous fibrous layer thickness and amount of Type I collagen in tails of mice treated with Linba Fang were significantly lower than those of negative and positive controls. Among mice treated with the same doses of Linba Fang, these effects were more significant in mice treated for 4 weeks than mice treated for 2 weeks. We also found that the effects of Linba Fang were positively correlated with its dose. These results demonstrated that Linba Fang possessed a strong therapeutic effect against fibrosis associated with lymphedema.

*Salvia miltiorrhiza*, another main component of Linba Fang, is commonly used to improve blood circulation and has pharmacological effects of inhibiting platelet aggregation, promoting fibrinolysis, improving blood capillary tone, improving microcirculation, reducing the fragility of blood capillaries, and reducing blood viscosity. In addition, *Salvia miltiorrhiza* contains large amounts of Tanshinone IIA, which can increase the expression of the Ero1-L*α* and DsbA-L gene and inhibit the expression of PPAR-*γ* gene [21], leading to decreased lipid deposition [22, 23]. In this study, we found that the thickness of the subcutaneous fat and levels of lipid deposition regulatory proteins (PPAR-*γ* and CEPB-*α*) in tails of mice treated with Linba Fang were significantly lower than negative and positive controls. Moreover, the effects of Linba Fang were positively correlated with its doses. These results demonstrated that Linba Fang also had the effect of inhibiting lipid deposition. In addition, Linba Fang could reduce the tail diameters and weights of mice with lymphedema significantly, and the effects were time- and dose-dependent. These results demonstrated that Linba Fang was effective against edema. Probable explanations are that Linba Fang can inhibit the pathological changes caused by lymphatic obstruction, including inflammation, fat deposition, and fibrosis, and reduce swelling and associated complications.

The main limitation of this study was that High-Performance Liquid Chromatography (HPLC) was not performed to determine biological fingerprinting of Linba Fang. In the next step, we will perform HPLC for the identification.

## 5. Conclusions

In summary, Linba Fang, consisting of Sophora flavescens Ait and *Salvia miltiorrhiza*, was effective against edema, inflammation, fibrosis, and lipid deposition.

## Figures and Tables

**Table 1:** Grouping and corresponding treatment of C57BL/6 mice.

Grouping	*n*	Establishment of the lymphedema model	Treatment	Treatment duration
Normal group	11	No	No	
Model 2	11	Yes	0.5% sodium carboxymethyl cellulose solution	2 weeks
Model 4	11	Yes	0.5% sodium carboxymethyl cellulose solution	4 weeks
AES-2	11	Yes	13.5 mg/kg aescuvenforte	2 weeks
AES-4	11	Yes	13.5 mg/kg aescuvenforte	4 weeks
MATL-2	11	Yes	500 mg/kg Linba Fang	2 weeks
MATM-2	11	Yes	1000 mg/kg Linba Fang	2 weeks
MATH-2	11	Yes	2000 mg/kg Linba Fang	2 weeks
MATL-4	11	Yes	500 mg/kg Linba Fang	4 weeks
MATM-4	11	Yes	1000 mg/kg Linba Fang	4 weeks
MATH-4	11	Yes	2000 mg/kg Linba Fang	4 weeks

## Data Availability

All data generated or analysed during this study are included in this published article.
